# Optimizing Hospital Electronic Prescribing Systems: A Systematic Scoping Review

**DOI:** 10.1097/PTS.0000000000000867

**Published:** 2021-05-14

**Authors:** Jac Williams, Stephen Malden, Catherine Heeney, Matt Bouamrane, Mike Holder, Uditha Perera, David W. Bates, Aziz Sheikh

**Affiliations:** From the ∗ Usher Institute, University of Edinburgh, Edinburgh, United Kingdom; † Division of General Internal Medicine and Primary Care, Brigham and Women’s Hospital, Boston, Massachusetts.

**Keywords:** patient safety, quality, efficiency, ePrescribing, health IT, medicines management

## Abstract

Considerable international investment in hospital electronic prescribing (ePrescribing) systems has been made, but despite this, it is proving difficult for most organizations to realize safety, quality, and efficiency gains in prescribing. The objective of this work was to develop policy-relevant insights into the optimization of hospital ePrescribing systems to maximize the benefits and minimize the risks of these expensive digital health infrastructures.

**Methods:**

We undertook a systematic scoping review of the literature by searching MEDLINE, Embase, and CINAHL databases. We searched for primary studies reporting on ePrescribing optimization strategies and independently screened and abstracted data until saturation was achieved. Findings were theoretically and thematically synthesized taking a medicine life-cycle perspective, incorporating consultative phases with domain experts.

**Results:**

We identified 23,609 potentially eligible studies from which 1367 satisfied our inclusion criteria. Thematic synthesis was conducted on a data set of 76 studies, of which 48 were based in the United States. Key approaches to optimization included the following: stakeholder engagement, system or process redesign, technological innovations, and education and training packages. Single-component interventions (n = 26) described technological optimization strategies focusing on a single, specific step in the prescribing process. Multicomponent interventions (n = 50) used a combination of optimization strategies, typically targeting multiple steps in the medicines management process.

**Discussion:**

We identified numerous optimization strategies for enhancing the performance of ePrescribing systems. Key considerations for ePrescribing optimization include meaningful stakeholder engagement to reconceptualize the service delivery model and implementing technological innovations with supporting training packages to simultaneously impact on different facets of the medicines management process.

In most health systems globally, replacing traditional paper-based processes and pathways with digital systems and services is now considered a strategic priority to modernize and optimize the efficiency and safety of healthcare. Previous implementation experiences have highlighted that this is usually a complex long-term process, involving multiple stakeholders, with different expectations and priorities.^1^ Electronic prescribing (also known as computerized physician order entry [CPOE] with or without computerized decision support [CDS] and henceforth referred to as ePrescribing) is an area of health-system digitization, which can provide a range of benefits—from more efficient medication ordering and administration processes to alerting, error prevention, and improved patient safety.^2^ ePrescribing systems also have the potential to promote prescriber adherence with evidence-based guidelines, facilitate cost-conscious prescribing, and enable changes in the medicines use process.^3^

Realizing the benefits of ePrescribing is largely dependent on optimizing these systems, so that the available functionalities are fully enabled, appropriately used, integrated with other relevant health information technology (IT), and embedded with clinical priorities and workflows. Substantial and cost-effective reductions in clinically important medication errors can be achieved with the implementation of ePrescribing systems, but these are not guaranteed. Successful deployment is contingent on the context of the ePrescribing implementation, and the use of identical software can lead to very different results in different hospitals, in part because these systems are highly configurable at the local level.^4^ ePrescribing systems are becoming increasingly complex, requiring iterative development and commitment to a life-cycle perspective.^5^ Emergent research focuses on ensuring these large-scale and expensive health IT infrastructures now deliver the promised clinical improvements through the process of systems optimization.^6^

The aim of this work was to map the landscape of optimization strategies within the field of hospital ePrescribing and develop an evidence-informed policy-focused overview of the range of approaches that have been deployed to maximize the patient, provider, and organizational benefits of ePrescribing systems.

## METHODS

### Overview of Methods

We used the 6-stage framework proposed by Arksey and O’Malley^7^ and then further refined by Levac et al^8^ to undertake a systematic scoping review of the international literature. Our methods are detailed in full in the published protocol and summarized hereinafter.^9^ Embracing the iterative nature of conducting a scoping protocol, a feature strongly endorsed by Levac et al,^8^ the inclusion and exclusion criteria from the initial protocol have been refined (Table 1). An essential requirement for included studies was the implementation of an optimization strategy within an ePrescribing system. Systems optimization was defined as “the activity of enhancing system capabilities and integration of subsystem elements to the extent that all components operate at or above user expectations.”^10^ ePrescribing was defined as “the utilization of electronic systems to facilitate and enhance the communication of a prescription or medicine order, aiding the choice, administration and supply of a medicine through knowledge and decision support, and providing a robust audit trail for the entire medicines use process.”^11^

**TABLE 1:** Inclusion and Exclusion Criteria

Inclusion criteria
Primary studies or systematic reviews with a clearly defined methodology that describe an approach/approaches that are implemented to optimize an ePrescribing system.
The study must take part in a healthcare context that is applicable to learning for UK NHS hospitals.
The study should be set in a high-income country, as defined by the OECD.
Exclusion criteria
Study does not describe an optimization strategy implemented in an ePrescribing system.
The study is an opinion piece or a review without a clearly defined methodology.
Study takes place in a healthcare context that is not applicable to learning for UK NHS hospitals.
The country of the study is not within the OECD.

OECD, Organization for Economic Co-operation and Development; NHS, National Health Service.

### Systematic Scoping Review

Abstracts and full texts were independently screened by 2 reviewers, with conflicts moderated by a third member of the research team (J.W., S.M., C.H., M.B., M.H., U.P.). In view of the substantial body of evidence uncovered, we decided to focus on the most recently published articles using principles of data saturation. Saturation is a methodological principle taken from qualitative research where it is mostly used as a criterion for discontinuing data analysis or collection.^12^ Although the concept of saturation still seems to be evolving, we identify with the 2016 definition by Given,^13^ who considered saturation as the point at which “additional data do not lead to any new emergent themes.” This involved conducting full-text screening (J.W., S.M., C.H., M.B.) and data extraction (J.W., S.M., M.B.) where we added a code for every new optimization strategy encountered during extraction. It was agreed that saturation would be achieved when no new codes emerged for optimization strategies from 10 consecutive studies.

### Interpretation of Findings

**FIGURE 1:**
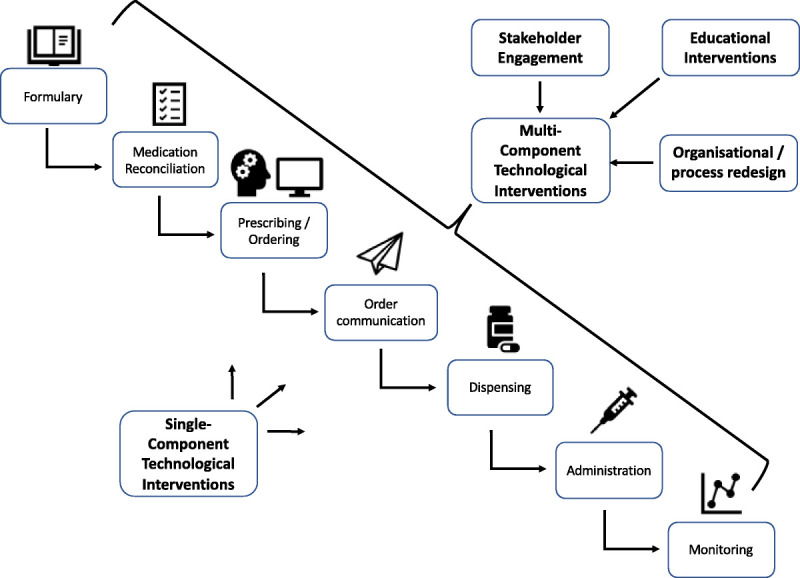
Theoretical approach showing a conceptualization of the medicines use process and the various stages with potential for optimization within an ePrescribing system.

Extracted data were synthesized by applying thematic analysis to a theoretical approach that considered the medication management life cycle and the propensity for errors at each stage of this process.^14^ ePrescribing optimization strategies were then thematically analyzed based on where the intervention lay within the ePrescribing process (Fig. 1).

Consultative phases with domain experts and patient and public involvement representatives were integrated throughout this work. The feedback from 2 round-table events with international and UK experts on ePrescribing, which took place in June 2019 and January 2020, were used to discuss and refine the thematic synthesis applied to our results.

## RESULTS

### Included Studies

**FIGURE 2:**
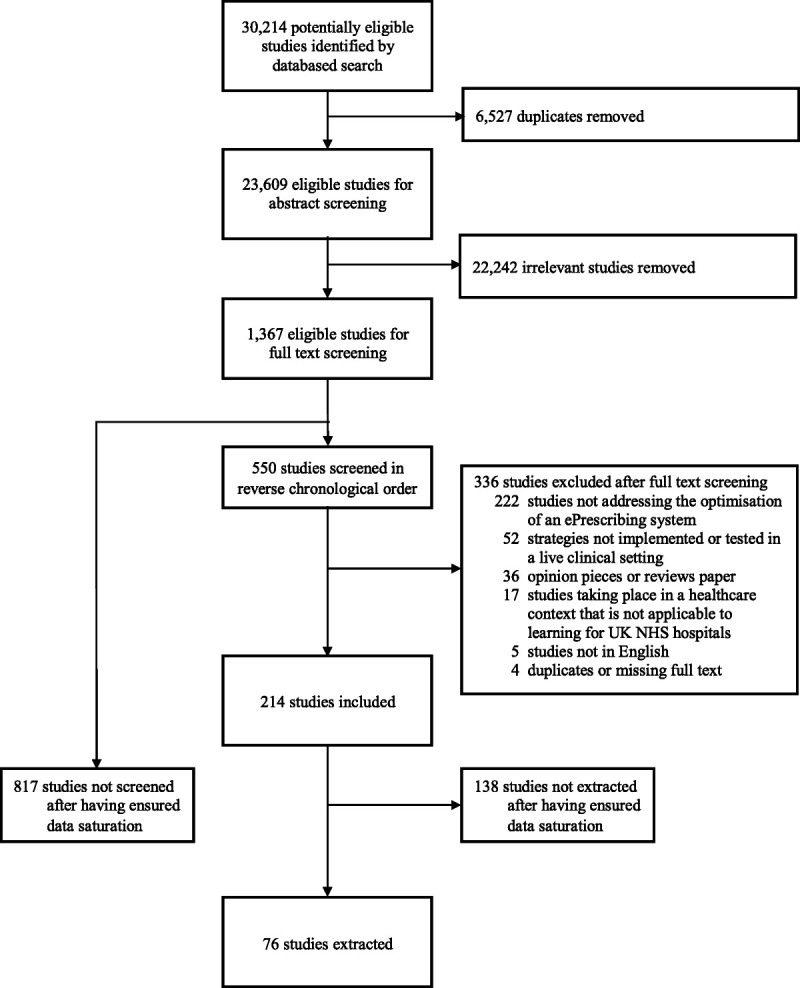
Modified Preferred Reporting Items for Systematic Reviews and Meta-Analyses diagram.

We identified 30,214 potentially eligible studies, which, after the removal of duplicate studies, resulted in 23,609 studies eligible for abstract screening. The abstract screening stage led to the identification of an initial set of 1367 records eligible for full-text screening. Studies were then screened and extracted in reverse chronological order, with 817 studies not screened because of reaching data saturation. Of the 550 studies screened, we excluded 336 leaving 214 included studies (Fig. 2).

**FIGURE 3:**
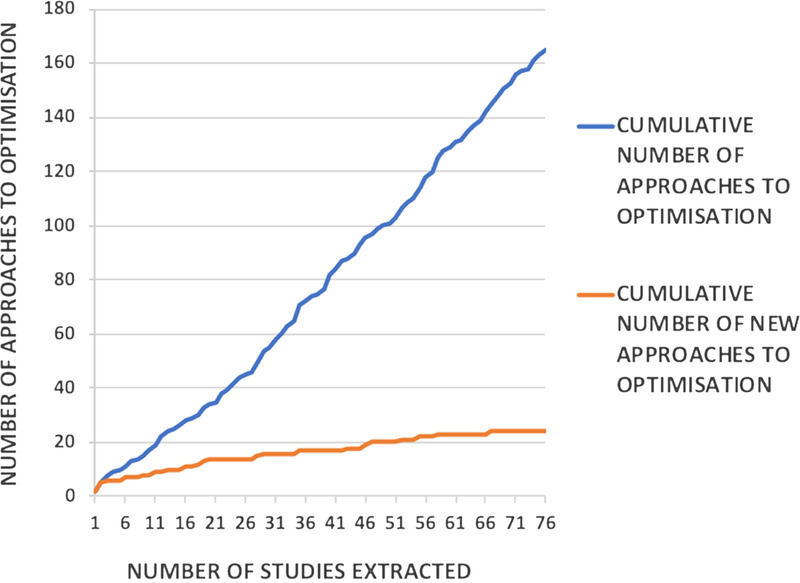
Saturation of new approaches to optimization when extracting data from the most recently published studies. Data extraction was discontinued after extracting 76 articles, when it seemed that new approaches to optimization were no longer emerging.

We applied criteria of inductive thematic saturation to the set of included studies, until no new optimization approaches were identified. We considered saturation to have been achieved when 10 consecutive studies did not yield any new approaches to optimization. Data extraction was accordingly discontinued after extracting 76 articles, published in the period from November 01, 2017, to November 06, 2019, when no new approaches to optimization emerged from 10 consecutive studies (Fig. 3).

### Characteristics of Included Studies

**FIGURE 4:**
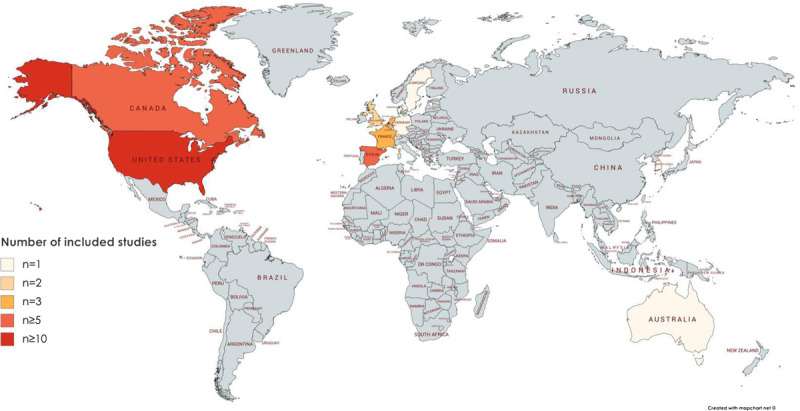
Heat map of the world showing countries based on the frequency of included studies.

The majority of the 76 included articles described U.S.-based studies (n = 48), with studies originating from Spain coming a distant second (n = 6; Fig. 4).

Optimization strategies implemented in the hospital setting (n = 61) accounted for most included studies and ranged from hospital-wide strategies (n = 29) and strategies encompassing multiple wards and departments (n = 12) to highly targeted, specialty-specific interventions (n = 20). A small number of studies were based on the optimization of ePrescribing systems in community healthcare (n = 13). A further study was conducted in both the hospital and community, and another was conducted in a clinical research setting.

Of the studies providing sufficient detail, the ePrescribing landscape was dominated by commercial ePrescribing software systems with Epic being the most frequently reported (n = 11) system. Many optimization strategies were reliant on the addition of third-party software packages or apps (n = 24).

Some studies related to single optimization strategies taking place at a very specific stage of the prescribing process. In contrast, other studies combined a number of optimization strategies, working in synergy in an attempt to achieve prescribing goals and support clinical outcomes. These can thus be viewed as multicomponent interventions, using multiple technological optimization strategies or by supplementing technological interventions with other strategies, such as educational interventions, stakeholder engagement, and organizational transformation.

### Single-Component Interventions

A number of studies (n = 26) described optimization strategies, implemented as single-component interventions (Table 2).^15–40^ Table 2 summarizes the various single-component optimization strategies used in the included studies, highlighting the stage of the ePrescribing process being optimized and the overall reported effectiveness of the intervention on the primary outcome.

**TABLE 2:** Single-Component Interventions Classified According to the Aspect of the ePrescribing Process Being Optimized

Stage of the ePrescribing Process Being Optimized	Study	Country	Single-Component Intervention	Study Design/Evaluation Method	Care Setting	Study Context	Primary Outcome Measure	Effect of Intervention
Formulary	Gayoso-Rey et al^15^ (2020)	Spain	Formulary changes	Prospective study	Secondary care	Standardizing an oncology drug database to reduce prescribing errors	Rate of prescribing errors	+
Medication reconciliation	Jurado et al^16^ (2018)	France	Transferring ePrescribing data across boundaries of care	Pilot study	Secondary care	Suitability of new medication reconciliation tool when patients admitted to hospital	Establishing an accurate medication profile	+
Prescribing/ordering	Epstein et al^17^ (2019)	Israel	CDSS	Pre/post study	Secondary care	The impact of CDSS in COPD management after discharge	Rate of adherence with discharge recommendations/guidelines	+
	Daniels et al^18^ (2019)	United States	Refining alerts	Quality improvement report	Secondary care	Improving drug-drug interaction alert effectiveness	No. interruptive drug-drug interaction alerts	+
	Wai et al^19^ (2019)	United States	CDSS	Retrospective cohort study	Secondary care	Improving correct prescribing of thiamine in patients with alcohol use disorder	Proportion of patients receiving high-dose parenteral thiamine	+
	Gupta et al^20^ (2019)	Canada	CDSS	Interrupted time series	Primary care	Improving asthma management in primary care	Proportion of patients with written asthma action plan	+
	Orchard et al^21^ (2018)	Australia	CDSS	Pre/post study	Primary care	Improving guideline-recommended therapy in atrial fibrillation	Proportion of patients appropriately prescribed anticoagulants	+
	Figueroa et al^22^ (2019)	Spain	Refining alerts	Pre/post study	Secondary care	Improving appropriate pharmacological prophylaxis	Prescribing of pharmacological prophylaxis	+
	Gaweda et al^23^ (2018)	United States	CDSS	Retrospective case-control study	Secondary care	Improving anemia management in dialysis patients	Percentage of hemoglobin concentrations between 10 and 12 g/dL	+
	Akhloufi et al^24^ (2018)	The Netherlands	CDSS	Mixed methods	Secondary care	Using CDSS to support intravenous to oral antibiotic switch	Clinical relevance and usefulness of CDSS	N/A
	Pendharkar et al^25^ (2018)	Canada	Order set	Step-wedge study	Tertiary care	Improving management of acute exacerbation of COPD	Hospital length of stay	/
	Simpao et al^26^ (2018)	United States	Data dashboard	Quality improvement report	Tertiary care	Developing an electronic antibiogram	No outcome measure—descriptive/feasibility report	N/A
Order communication	Choi et al^27^ (2019)	Korea	Prescription validation/intervention	Retrospective study	Secondary care	Impact of pharmacist interventions on physicians’ acceptance of CDSS recommendations	The no. dosing alerts and physicians’ acceptance rates	+
	Jourdan et al^28^ (2018)	France	Prescription validation/intervention	Prospective study	Secondary care	Impact of pharmacist interventions on clinical outcome and cost	No. avoided hospitalization days and associated cost avoidance	+
	Groppi et al^29^ (2018)	United States	Prescription validation/intervention	Descriptive research report	Secondary care	Streamlining the documentation process to capture pharmacist interventions	No outcome measure—descriptive/feasibility report	N/A
	Durvasula et al^30^ (2018)	United States	Alerts	Retrospective study	Secondary care	High-cost medications triggered an electronic alert to a review committee	Retrospective cost savings	+
Medication dispensing	Berdot et al^31^ (2019)	France	Robotic dispensing technology	Mixed methods	Secondary care	Evaluation of an automated-dispensing system after implementation and upgrade	Return on investment and quality improvement metrics	+
	Lupi et al^32^ (2020)	United States	Automated dispensing cabinet	Pre/post study	Tertiary care	Optimization of automated dispensing cabinets by clinical pharmacists	No. dispenses from central pharmacy, no. stockouts, inventory cost	+
	Campmans et al^33^ (2018)	The Netherlands	Alerts	Cross-sectional study	Community pharmacy	Reducing medication dispensing errors	Evaluating the experience of pharmacists using a survey	N/A
	Rodriguez-Gonzalez et al^34^ (2019)	Spain	Robotic dispensing technology	Pre/post study	Tertiary care	Impact of implementing a robotic dispensing system	Frequency of medication dispensing errors	+
	Beobide Telleria^35^ (2018)	Spain	Automated tablet dispensing and packaging system	Pre/post study	Nursing home	Impact of implementing an ATDPS	No. medication dispensing errors	+
	Bhakta et al^36^ (2018)	United States	Automated robotic compounding technology	Quasi-experimental study	Tertiary care	Impact of implementing ARCT in a satellite oncology pharmacy	Turnaround time for preparations	+
Medication administration	Campbell et al^37^ (2018)	United States	Data dashboard	Pre/post study	Secondary care	Implementing a dashboard allowing nurses to visualize their individual near-miss medication error	No. near-miss medication administration events	+
Monitoring	Pereboom et al^38^ (2019)	The Netherlands	CDSS	Pre/post study	Secondary care	Improving adequate dosing of gentamicin and vancomycin	Plasma concentrations measured within 72 h	+
	Beeler (2019)^39^	Switzerland	CDSS	Cluster randomized control trial	Tertiary care	The impact of CDSS in potentially serious potassium-increasing drug-drug interactions	The frequency of potassium-monitoring intervals >72 h	/
	Kim (2018)^40^	United States	CDSS	Post hoc analysis	Research setting	Using pharmacogenetic testing to identify drug therapy problems in polypharmacy patients	No. drug therapy problems per patient	/

+ = intervention significantly improved primary outcome; / = intervention effects were null.

ATDPS, automated tablet dispensing and packaging system; ARCT, automated robotic compounding technology; CDSS, computerized decision support system; N/A, intervention did not measure objective outcomes.

All of the single-component optimization strategies were technological interventions targeting a single step in the prescribing process. The optimization strategies most frequently implemented were those at the prescribing interface (n = 10), and the most frequently deployed single-component optimization strategy was CDS systems (n = 9).

#### Formulary Changes

In 1 study, targeting formulary changes, active pharmaceutical ingredients in the electronic formulary of a Spanish hospital were standardized and implemented within an oncology outpatient clinic.^15^ This optimization work was associated with a decrease in medication errors. One of the key factors in the work was the implementation of “tall-man letters” in the electronic prescribing system with the aim of reducing errors relating to similarly named medications (e.g., DOBUTamine and DOPamine).

#### Medication Reconciliation

Medication reconciliation represents a crucial step in the prescribing process for hospitalized patients that aims to reduce the risk of iatrogenic harm through a comprehensive review of patients’ medication history. A study conducted in a hospital diabetes service in Toulouse, France, used the Electronic Pharmaceutical Record to summarize patients’ medication history, including over-the-counter drugs, dispensed by community pharmacies over the previous 4 months.^16^ By using national health insurance card details and linking to a secure Electronic Pharmaceutical Record server, the study reported improved medication reconciliation accuracy. In almost 30% of patients, medication errors that had not previously been identified were able to be detected and rectified.

#### Prescribing

At the prescriber interface of ePrescribing systems, CDS,^17,19–21,23,24^ order sets,^25^ alerts,^18,22^ and a data dashboard^26^ were all deployed as optimization strategies. Computerized decision support was often applied as an optimization strategy to aid the management of clinical conditions with standardized treatment algorithms amenable to decision support.^17,19–21^ Computerized decision support was also used for broader clinical goals, such as to promote switching of antibiotics from the intravenous to oral route to support antimicrobial stewardship,^24^ and for anemia management in dialysis patients.^23^

Reflecting the increasing body of literature around alert fatigue, and the risks associated with this optimization strategy, some studies attempted to optimize the existing alerts within their ePrescribing systems.^18,22^ For example, Daniels et al^18^ sought to optimize drug-drug interaction alerts at a pediatric hospital in Tennessee either through suppression or by using patient-specific data from the electronic health record to contextualize and filter alerts. The number of interruptive drug-drug interaction alerts was reportedly reduced by 40% for all clinicians.

#### Order Communications

A frequently deployed strategy was to empower pharmacists to intercept and validate prescriptions at the order communication stage (n = 3). Some of the studies applied this intervention as a second check to capture errors and thereby improve the quality and safety of prescribing,^27–29^ whereas a couple of the studies used this strategy to reduce medication costs.^28,30^

A caveat to relying on assessment of prescriptions is the substantial volume of electronic prescriptions that are ordered throughout hospitals on a daily basis. To tackle this issue, Jourdan et al^28^ used a third-party software called PHARMA to identify specific high-risk medications or restricted usage drugs for pharmacist review. By optimizing the pharmacist review process, the research group estimated that they would have prevented 73 intensive care admission days, 74 continuous monitoring unit hospitalization days, and 66 days of conventional hospitalization over the 6-month study period. This equated to €5.09 of public health savings for every euro invested in the prescription review activity.

#### Dispensing

Single-component interventions optimizing the medication dispensing process were dominated by large capital expenditures in robotics and automation, namely, automated dispensing cabinets,^32^ robotic dispensing technology,^31,34^ automated tablet dispensing and packaging systems,^35^ and automated robotic compounding technology.^36^ These studies mainly focused on increased efficiency and cost savings as well as improving patient safety by reducing medication dispensing errors.

A significant reduction in dispensing errors was achieved by implementing centralized robotic dispensing systems in hospital pharmacies^31,34^ and by installing an automated tablet dispensing and packaging system in 7 Spanish geriatric nursing homes.^35^ Cost savings were achieved^32^ at an 800-bed academic medical center by reducing the inventory cost across 65 automated dispensing cabinets. Through careful analysis and optimization of the medications held in the automated dispensing cabinets, they were also able to reduce the number of out of stock medications and the number of dispenses from central pharmacy. Meanwhile, implementing automated robotic compounding technology in a satellite oncology pharmacy significantly decreased the turnaround time for medications, and a return on investment within 8.6 years was projected through supply cost savings.^36^

#### Administration

To optimize medication administration, Campbell et al^37^ used real-time data, extracted from the electronic health record, to warn nurses of their individual “near-miss medication error risk” delivered through a visual dashboard. By correlating near-miss medication errors reported through the Barcode Medication Administration System with environmental data related to call lights, patient count, medication count, hours worked, sepsis scores, and task count for a 25-bed unit, the authors performed a regression analysis to identify factors predisposing to errors on an individual practitioner and unit-wide basis. During this pilot study, the intervention reported a reduction in near-miss events.

#### Monitoring

Optimization strategies for monitoring prescribed medications attempted to identify and reduce adverse drug reactions^40^ or drug-drug interactions.^39,40^ In 1 study, CDS was combined with pharmacogenetic data to help identify drug therapy problems in polypharmacy patients,^40^ whereas in another study, 5 features of an existing CDS were optimized to deliver patient-specific decision support for potassium-increasing drug-drug interactions. Neither of these studies were reported as having a positive effect on their respective primary end points. In contrast, Pereboom et al^38^ reported successfully using CDS to improve plasma concentration monitoring, thereby supporting appropriate dosing of antibiotics with pharmacokinetic dosing rules, such as gentamicin and vancomycin.

### Multicomponent Interventions

Most studies (n = 50) reported on interventions that used more than 1 approach to optimize ePrescribing systems (Table 3).^4,41–94^ Table 3 summarizes the various combinations of optimization strategies and intervention components that were used in the included studies and the overall reported effectiveness of the intervention on the primary outcome.

**TABLE 3:** Multicomponent Interventions Classified According to the Combination of Intervention Strategies Deployed

Optimization Strategies Used	Study	Country	Specific Intervention Components	Study Design/Evaluation Method	Care Setting	Stage of ePrescribing Process Being Optimized	Primary Outcome Measure	Effect of Intervention
1	Burkoski et al^41^ (2019)	Canada	BCMA, closed-loop medication system	Interrupted time series	Secondary care	Whole prescribing process	Medication errors and adverse drug events	+
	Pettit et al^42^ (2019)	United States	CDSS, CPOE	Pre/post study	Secondary care	Prescribing/ordering	Medication prescribing errors	+
	Bowdle et al^43^ (2018)	United States	BCMA, CDSS, smart infusion pumps	Pre/post study	Secondary care	Prescribing/ordering, dispensing, administration	Medication administration errors	+
	Ashburner et al^44^ (2018)	United States	Alerts, CDSS	Randomized controlled trial	Primary care	Prescribing/ordering	Medication prescribing rates	/
	Risor (2018)^45^	Denmark	Automated dispensing cabinets, BCMA, CPOE	Pre/post study	Secondary care	Administration	Medication administration errors	/
	Schnock et al^46^ (2018)	United States	BCMA, smart infusion pumps	Pre/post study	Secondary care	Administration	Medication administration errors	+
	Desmedt et al^47^ (2018)	Belgium	Alerts, CDSS	Pre/post study	Secondary care	Prescribing/ordering	Prescription dose appropriateness	/
	Karlsson et al^48^ (2018)	Sweden	Alerts, CDSS	Cluster randomized controlled trial	Primary care	Prescribing/ordering	Adherence to prescribing guidelines	+
	Gunn et al^49^ (2018)	United States	Alerts, CDSS indication-based prescribing	Prospective cohort study	Secondary care	Prescribing/ordering	Provider-initiated medication order	+
	Macias et al^50^ (2018)	Spain	BCMA, CPOE	Pre/post study	Tertiary care	Administration	Medication administration errors	+
	Ni et al^51^ (2018)	United States	BCMA, CDSS, drug monitoring, refining alerts, smart infusion pumps	Prospective cohort study	Tertiary care	Administration, monitoring	Detection of dosing-related medication administration errors	+
	Rosa et al^52^ (2018)	United States	Alerts, order set	Interrupted time series	Secondary care	Prescribing/ordering, administration	Compliance and timing of care	+
1, 2	Ilcewicz et al^53^ (2019)	United States	Order set, prescriber education	Retrospective cohort study	Secondary care	Prescribing/ordering, administration, monitoring	Glycemic control over 72 h	+
	MacMaster et al^54^ (2019)	United States	Automated dispensing cabinet, BCMA, nurse education	Observational study	Secondary Care	Administration	Medication administration errors	+
	Thompson et al^55^ (2018)	United States	BCMA, staff education	Pre/post study	Secondary care	Administration	Medication administration errors	+
	Mathioudakis et al^56^ (2018)	United States	CDSS, prescriber education	Quality improvement study	Secondary care	Prescribing/ordering	Optimization design process	N/A
	Mainous et al^57^ (2018)	United States	CDSS, prescriber education	Pre/post study	Primary care	Prescribing/ordering	Iron test ordering	+
	Gulati et al^58^ (2018)	United States	Order set, prescriber education	Retrospective cohort study	Secondary care	Prescribing/ordering	Cumulative steroid use	+
	O’Sullivan et al^59^ (2018)	United States	CDSS, prescriber education	Retrospective cohort study	Secondary care	Prescribing/ordering	Compliance rate with intraoperative antibiotic redosing criteria	+
	Connor et al^60^ (2018)	United States	CDSS, data dashboard, prescriber education	Pre/post study	Secondary care	Prescribing/ordering	Single-unit blood transfusion rates	+
	Tamblyn et al^61^ (2018)	Canada	CPOE, data dashboard, prescriber education	Pre/post study	Tertiary care	Whole prescribing process	Medication reconciliation completion rates	+
	Pontefract et al^4^ (2018)	United Kingdom	CDSS, CPOE	Pre/post study	Secondary care	Prescribing/ordering	Medication prescribing errors	N/A
1, 3	Muhlenkamp et al^62^ (2019)	United States	Refining alerts, stakeholder engagement	Pre/post study	Secondary care	Prescribing/ordering	Change in dosage alerts	+
	Kawamanto^63^ (2018)	United States	CDSS, refining alerts, stakeholder engagement	Pre/post study	Secondary care	Prescribing/ordering	Alert appropriateness	+
	Crespo et al^64^ (2018)	Canada	CDSS, stakeholder engagement	Multicenter observational study	Secondary care	Whole prescribing process	Identification of medication discrepancies	N/A
1, 4	Gill et al^65^ (2019)	United States	CDSS, prescriber feedback	Prospective randomized study	Primary care	Monitoring	Hemoglobin A1C levels	+
	Quintens et al^66^ (2019)	Belgium	CDSS, prescription validation/intervention	Retrospective cohort study	Secondary care	Whole prescribing process	No. alerts resulting in pharmacist intervention, physician acceptance rates	+
	Carver et al^67^ (2018)	United States	Alerts, CDSS, prescription validation/intervention	Multicenter retrospective study	Secondary care	Whole prescribing process	Incidence of IV to oral therapy conversion, associated cost savings	+
	Kang et al^68^ (2018)	United States	Alerts, CDSS, prescription validation/intervention	Pre/post study	Secondary care	Prescribing/ordering, order communication	Formulary medication utilization	+
	Christ et al^69^ (2018)	United States	CDSS, prescription validation/intervention	Pre/post study	Secondary care	Prescribing/ordering, order communication	Attainment of analgesia at 24 h from admission	/
	Amor-Garcia^70^ (2018)	Spain	CPOE, prescription validation/intervention	Pre/post study	Secondary care	Prescribing/ordering, order communication	Implementation of additional pharmacist checks and CPOE	N/A
	Howell et al^71^ (2019)	United States	CDSS, prescription validation/intervention	Prospective study	Secondary care	Whole prescribing process	No. alerts, no. interventions	N/A
	Lesselroth et al^72^ (2018)	United States	Data dashboard, drug monitoring, prescription validation/intervention	Randomized controlled trial	Primary care	Monitoring, order communication	Medication reconciliation rates	/
	Horng^73^ (2018)	United States	Automated dispensing cabinet, CPOE, prescription validation/intervention	Pre/post study	Secondary care	Dispensing, administration, order communication	Time from antibiotic order entry to medication administration	+
	Peters-Strickland et al^74^ (2018)	United States	Data dashboard, drug monitoring, prescriber feedback	Human factor/validity study	Primary care	Monitoring	Performance task failure rates	+
	Harvin et al^75^ (2018)	United States	Data dashboard, prescription validation/intervention, drug monitoring	Pre/post study	Secondary care	Order communication	Time to intervention	+
	Kummer et al^76^ (2018)	United States	Order set, transferring ePrescribing data across boundaries of care	Pre/post study	Tertiary care	Whole prescribing process	Administration of tissue plasminogen activator	+
1–3	Biltoft et al^77^ (2018)	United States	Smart infusion pumps, prescriber education, stakeholder engagement	Case study	Secondary care	Administration	Patient safety, revenue-generation gains	+
1, 2, 4	Gabel et al^78^ (2019)	United States	CDSS, prescriber education, prescriber feedback	Pre/post study	Secondary care	Prescribing/ordering, administration	Incidence of postoperative nausea and vomiting	+
	Nguyen et al^79^ (2020)	United States	CDSS, prescriber education, prescription validation/intervention	Quality improvement study	Secondary care	Prescribing/ordering, order communication	Monthly spending on intravenous acetaminophen	+
	Gulliford et al^80^ (2019)	United States	CDSS, prescriber education, prescriber feedback	Cluster randomized controlled trial	Primary care	Prescribing/ordering	Rate of antibiotic prescriptions for respiratory tract infections	+
	Adeola et al^81^ (2018)	United States	Alerts, CDSS, order set, formulary changes, prescriber education	Pre/post study	Tertiary care	Prescribing/ordering	Exposure to target medications	+
	Gong et al^82^ (2019)	United States	CDSS, mandatory free-text justification of a prescription, prescriber feedback, prescriber education	Longitudinal study	Primary care	Prescribing/ordering	Cost-effectiveness and QALYs	+
	Shea et al^83^ (2018)	United States	Alerts, formulary changes, pharmacist education, prescription validation/intervention	Retrospective cohort study	Secondary care	Whole prescribing process	Medication error rate	+
	Muluneh et al^84^ (2018)	United States	Calculating adherence using medication possession ratio, closed-loop medication system, patient education, prescription validation/intervention	Pre/post study	Tertiary care	Whole prescribing process	Patient knowledge tests, adherence, molecular response rate	+
	Muth et al^85^ (2018)	United States	CDSS, Prescriber education, Prescription validation/intervention	Cluster randomized controlled trial	Primary care	Whole prescribing process	Mean Medication Appropriateness Index sum score	/
	Peyko et al^86^ (2018)	United States	Drug monitoring, prescriber and pharmacist education, prescription validation/intervention	Pre/post study	Secondary care	Prescribing/ordering, order communication	Timing of vancomycin level assessment	+
1–4	Lane et al^51^ (2019)	United States	Data dashboard, staff and patient education, stakeholder engagement	Case study	Tertiary care	Whole prescribing process	Implementation of an antimicrobial stewardship program	N/A
	Conners et al^87^ (2018)	United States	Order set, pay for performance bonuses, prescriber education, prescriber feedback, stakeholder engagement	Pre/post study	Secondary care	Prescribing/ordering	Asthma order set usage frequency	+
	Weiner et al^88^ (2019)	United States	Alerts, CDSS, order set, prescriber education, stakeholder engagement, transferring ePrescribing data across boundaries of care	Quality improvement study	Primary and secondary care	Whole prescribing process	No. opioid prescriptions per month	+

1 = technological intervention; 2 = educational intervention; 3 = stakeholder engagement; 4 = organizational/process redesign. + = intervention significantly improved primary outcome; / = intervention effects were null.

IV, intravenous; QALY, quality-adjusted life year; N/A, intervention did not measure objective outcomes.

Many multicomponent interventions targeted a single step in the prescribing process, such as drug prescribing/ordering, medication administration, medication dispensing, and monitoring. In contrast, other multicomponent interventions targeted multiple stages in the prescribing process. In 1 study, a multifaceted approach included changes to many aspects of the CPOE system, alongside integration of data from regional databases and substantial work on culture change through education and targeted meetings, involving both clinical and IT professionals.^88^ In other cases, the focus of the multilevel intervention was much narrower in scope, for example, by targeting changes in technical systems to support barcode medicines administration.^94^

A multitude of optimization strategies were adopted within these 50 multicomponent interventions. The specific components implemented varied considerably between studies; however, these could be broadly categorized into 4 distinct intervention strategies, namely, technological innovations (such as installation of automated dispensing equipment), educational packages (such as formal training sessions for prescribers/pharmacists), organizational/process redesign interventions (such as formulary changes and additional personnel procedures during the medication reconciliation process), and stakeholder engagement (consisting of involving relevant staff in the design and adaptation of specific intervention strategies). These strategies were used in different combinations within the identified studies.

Some form of technological innovation was deployed in every multicomponent study. Of these, CDS was the most commonly used technology (n = 27) often with alerts (n = 13). Although some studies implemented alerts as an optimization strategy, many also sought to refine and modify these to reduce alert fatigue. For example, Kawamoto et al^92^ targeted alert fatigue by refining alerts in Epic using stakeholder engagement to determine how best to alter alert functionality. Specifically, an expert governance group was consulted and high-frequency, low-value alerts were identified and disabled or modified. This resulted in reported significant reductions in alerts per visit and overall alert volume over the 3-year period from baseline to posttest. In addition, alerts leading to a discontinuation of the triggering medication within 1 hour increased by a reported 17%. Five studies implemented a technological intervention in conjunction with some form of stakeholder engagement. Additional technological innovations included barcode medication administration (BCMA, n = 8), CPOE (n = 7), order sets (n = 7), visualization tools/data dashboards (n = 6), smart infusion pumps (n = 4), computerized medication reconciliation software (n = 2), and closed-loop medication systems (n = 2).

Education was a consistently implemented strategy across the included studies, being applied in some form within 23 of the multicomponent interventions. Education often coincided with the implementation/optimization of new ePrescribing technology and focused on adequately training staff to use the new systems/software. One study, for example, used a strategy of training clinical staff, while at the same time supporting cultural change to empower pharmacists to enforce restrictions within the prescribing process.^79^ Again, in some cases, the educational component was focused on effecting organizational change, whereas, in one example, it was provided by the ePrescribing system vendor to enable clinical users of the system to manage changes to the CDS.^57^

Eighteen studies used some form of organizational/process redesign. As with interventions involving education or stakeholder engagement, organizational/process redesign interventions typically accompanied some form of technological intervention. Small-scale changes, such as pharmacist-led prescription validation procedures, comprised the majority of these studies. However, a small number of studies involved more innovative, larger-scale interventions, such as the implementation of an antimicrobial stewardship program,^51^ a closed-loop, pharmacist-led oral chemotherapy program,^84^ integration of ePrescribing systems across boundaries of care,^76,88^ or the addition of mandatory free-text justification for a medication.^82^ There were also some examples of changes to organizational data governance within existing ePrescribing systems because of the integration of data from national and regional databases. This occurred, for example, in the case of a national database of drug-drug interactions^66^ and a state-level database of prescription drug monitoring programs.^88^

Relatively few studies directly measured aspects of patient harm (such as adverse drug events or mortality; Kim, 2018^40^; Biltoft et al,^77^ 2018; Gabel et al,^78^ 2019). Instead, the majority used surrogate outcomes as proxy measures for patient safety or efficiency, such as medication error rates, hospital length of stay, blood sampling or timing of medication administration, among others (Tables 2, 3).

## DISCUSSION

### Principal Findings

There is now a substantial body of published work, mainly from the United States, that has targeted a range of medication management processes, using both relatively simple and more complex multifaceted interventions to enhance ePrescribing systems. Technological innovations comprised the majority of the strategies reported in the literature. However, technology-driven interventions were also often deployed in combination with behavioral components, including education, stakeholder engagement, and organizational change.

The fact that many studies are now focusing on refining alerts to reduce the burden of alert fatigue reflects the potential risks that can be introduced with digitization of prescribing processes. The healthcare context is also an important consideration for ePrescribing optimization strategies, with integrated healthcare systems aiding the detection of medication overuse, errors, and interactions. This is well illustrated by Jurado et al^16^ who used integrated data from community pharmacies to help detect medication discrepancies on admission to hospital and also Weiner et al^88^ who developed integrated information exchange between statewide emergency departments to counter opioid overuse. Conversely, nonintegrated healthcare systems will undoubtedly struggle to implement these optimization strategies.

Many strategies that successfully filter or contextualize data have shown evidence of success, particularly when such strategies focus on clinical impact, either by identifying high-risk patients or high-risk medications.^28^ Some studies have demonstrated that advanced capabilities can be achieved through the optimization of ePrescribing systems. The optimization of an ePrescribing system was central to the development of an antimicrobial stewardship program in an integrated healthcare system spanning many hospitals, with clinical decision support, data dashboards, and development of methods to track and report antimicrobial stewardship interventions. Likewise, highly integrated and contextualized environmental data from a ward setting allowed Campbell et al^37^ to provide real-time warnings to nursing staff about their risk of medication administration errors through a visual dashboard.

Achieving a return on investment through ePrescribing optimization was most frequently reported in the context of medication dispensing. Optimization with robotics and automation, whilst representing significant investments, seem to deliver a return on investment with quantifiable improvements to the safety and quality of the medication process.^31,32,34–36^

All of the single-component optimization strategies that we identified focused on technological interventions. This is likely to be a direct result of our search strategy and also our inclusion and exclusion criteria. Our searches combined broad terms, such as “quality improvement” and “audit”; however, our searches also focused on known optimization strategies, such as “CDS” and “alerts.” As such, to constitute optimization of an ePrescribing system, single interventions almost always had a technological aspect to ensure that the work was clearly relevant to ePrescribing systems. It is also possible that some of the single optimization strategies actually had more components to the intervention than were reported in the published study. For example, interventions may have been implemented with staff education; however, the educational component may not have been fully described. Of the articles that did describe the intervention, it was generally apparent that interventions with more components were also more likely to target multiple stages of the prescribing process, reflecting the complexities involved in optimizing numerous aspects of an ePrescribing system, and the need for a multifaceted approach.

Multicomponent interventions were overwhelmingly implemented within secondary care settings. Of these, all used either a quasi-experimental or observational study design, finding the intervention to be effective at optimizing the ePrescribing system in most studies (21/24 of studies measuring objective outcomes). Interestingly, results of multicomponent interventions within the primary care setting were more equivocal, with only 7 of the 10 studies reporting positive findings. Furthermore, all of the studies reporting null effects in the primary care setting were randomized controlled trials,^44,72,85^ possibly indicating that bias associated with nonrandomized and observational study designs may be inflating the observed effect estimates in secondary care settings.^94^ Inflated positive results, where randomization and blinding are not possible, also raise the possibility of the Hawthorne effect where subjects modify their behavior in response to being observed or measured. Conversely, it may be that the primary care setting presents barriers to the optimization of ePrescribing systems that are not present in secondary care. The primary care setting often provides continuity of care, with the reconciliation and arbitration of prescribed medications from a variety of other settings. The extent of integration and data sharing between hospitals, primary care, and community pharmacies within a healthcare system will either facilitate or impede the detection of medication errors and interactions. Where strong integration and transfer of data exist, optimization of ePrescribing systems may not alter what are already deemed to be well-managed patients and safe prescribing practices.^44,85^ However, as no formal appraisal of study quality was undertaken (as per usual practice in scoping reviews), it cannot be assumed that these results were not merely due to study quality issues, and the points discussed previously merit further investigation.

While a scarcity of experimental research was a finding of note in this review, so too is the abundant use of surrogate outcomes. The use of surrogate outcomes to determine the effectiveness of the included studies is unsurprising given the rarity of adverse events, which lead to clinically significant harm, coupled with the large sample sizes required to detect statistically significant changes to this outcome. However, although outcomes, such as medication error rates, do correlate relatively well with patient harm, the relationship between patient harm and other outcomes, such as efficiency of medication administration, or prescriber satisfaction is less clear. Therefore, interventions reporting positive results for such outcomes do not definitively demonstrate an improvement to patient safety and should be interpreted with caution.

### Strengths and Limitations

By following the principles of inductive thematic saturation,^13^ working backward chronologically, this has allowed the scoping review to succinctly map the most emergent and technologically relevant optimization strategies in this diverse field of literature. In this study, we describe a novel application of data saturation within a scoping review. Although we have carefully linked the concept of saturation to a central objective of the scoping review, namely, to describe the range of approaches to optimization, we acknowledge that by working backward through the most recently published literature, this may have introduced bias. By reaching saturation and only capturing the most recent optimization strategies, our scoping review could be missing fundamental optimization strategies in the ePrescribing journey that have been historically reported.

Assessing the impact of the various optimization strategies described in the literature is also challenging. Many studies measured the success of their interventions by using surrogate measures without addressing important clinical outcome measures. Even when studies did consider both process and outcome measures, improving ePrescribing processes with optimization strategies did not guarantee success in terms of clinical outcomes. For example, Figueroa et al^22^ successfully optimized ePrescribing alerts, resulting in improved prescribing of low molecular weight heparin; however, this did not have a significant impact on the incidence of venous thromboembolic events. Vice versa, Pendharkar et al^25^ reduced the length of acute hospital admissions in chronic obstructive pulmonary disease (COPD) patients but also reported a low uptake of the order set that was critical to their optimization strategy.

Another limitation of this scoping review is that optimization strategies that are easy to measure or that are amenable to research and publication will be overrepresented in the literature. This publication bias may have led to strategies that are difficult to measure or publish not appearing in the literature. We postulate that this may relate to some of the fundamental elements of system maintenance that may not be considered “worthy” of publication and also elements of optimization that are difficult to measure such as cyber security, software updates, and data storage and backup.

### Implications for Policy, Practice, and Research

Pharmacists, clinicians, nurses, hospital management, and health IT specialists are all key stakeholders in driving forward improvements in medicine management. Consulting and working with key stakeholders were identified in many studies as an important facilitator in the ePrescribing optimization journey.^87,88^ Pharmacists, as custodians of many of the stages within the medication use process, are rightly reflected in the literature as playing a central role in many of the optimization strategies encountered. Patients were, however, notably exempt as stakeholders—an oversight that will need to be addressed to ensure that the voices of the main beneficiaries are adequately heard.

It also seems that pharmacists, doctors, and nurses use ePrescribing systems as customizable tools to deliver localized and specialty-specific quality improvement aims. These optimization strategies can be powerful, achieving strong clinical buy-in and ownership, while also allowing ePrescribing systems and workflows to be customized extensively to local clinical and specialty-specific needs.^81^ Although localized innovation may be an effective method to improve usability and relevance of ePrescribing systems, optimization at scale will be dependent on success stories being cascaded and efficiently applied elsewhere. Poorly managed localized customization has the risk of leading to increasingly divergent systems and workflows, making policy deliberations and large-scale interventions difficult to manage. Policy-focused interventions will need to strike a balance between being sensitive to local needs, while delivering interventions that can drive tangible improvements in clinical outcome measures across large patient populations.

When extracting data, we aimed to link the optimization strategies being deployed with ePrescribing systems, third-party software, and apps. Unfortunately, the scoping review revealed a paucity of technical data and many studies neglected to name the ePrescribing software (n = 53) let alone describe the system specifications and capabilities. When describing changes, improvements, and optimization strategies relating to ePrescribing systems, authors should endeavor to name the software system and the version being used. Journals could play a role in mandating minimum levels of critical information and providing authors with appendices to document technical specifications.

## CONCLUSIONS

There is now substantial knowledge on approaches to optimize and potentially enhance the beneficial impacts of hospital ePrescribing systems. These include targeting single or multiple facets of the medicines management process. These approaches can be categorized as those focusing primarily on understanding and responding to stakeholder needs, reconceptualizing the medicines management process of care, technological innovations, and educational and training packages. Simultaneously deploying a combination of these approaches is likely to have the greatest impact on realizing the benefits of this increasingly ubiquitous technology.
